# Large Cryogenic Magnetostriction Induced by Hydrostatic Pressure in MnCo_0.92_Ni_0.08_Si Alloy

**DOI:** 10.3390/ma16031143

**Published:** 2023-01-29

**Authors:** Xiaowen Hao, Hongwei Liu, Bo Yang, Jie Li, Zhe Li, Zongbin Li, Haile Yan, Yudong Zhang, Claude Esling, Xiang Zhao, Liang Zuo

**Affiliations:** 1 Key Laboratory for Anisotropy and Texture of Materials (Ministry of Education), School of Material Science and Engineering, Northeastern University, Shenyang 110819, China; 2 Center for Magnetic Materials and Devices, Key Laboratory for Advanced Functional and Low Dimensional Materials of Yunnan Higher Education Institute, Qujing Normal University, Qujing 655011, China; 3 Laboratoire d’Étude des Microstructures et de Mécanique des Matériaux (LEM3), CNRS UMR 7239, Université de Lorraine, 57045 Metz, France; 4 Laboratory of Excellence on Design of Alloy Metals for Low-mAss Structures (DAMAS), Université de Lorraine, 57045 Metz, France

**Keywords:** magnetostriction, MnCoSi-based alloy, meta-magnetic transition, hydrostatic pressure, texture

## Abstract

Giant magnetostriction could be achieved in MnCoSi-based alloys due to the magneto-elastic coupling accompanied by the meta-magnetic transition. In the present work, the effects of hydrostatic pressure on magnetostrictive behavior in MnCo_0.92_Ni_0.08_Si alloy have been investigated. The saturation magnetostriction (at 30,000 Oe) could be enhanced from 577 ppm to 5034 ppm by the hydrostatic pressure of 3.2 kbar at 100 K. Moreover, under a magnetic field of 20,000 Oe, the reversible magnetostriction was improved from 20 ppm to 2112 ppm when a hydrostatic pressure of 6.4 kbar was applied at 70 K. In all, it has been found that the magnetostrictive effect of the MnCo_0.92_Ni_0.08_Si compound is strongly sensitive to external hydrostatic pressure. This work proves that the MnCoSi-based alloys as a potential cryogenic magnetostrictive material can be modified through applied hydrostatic pressure.

## 1. Introduction

Magnetostriction refers to the deformation of a crystalline solid induced by an applied magnetic field, and the technology is indispensable in sonars, energy harvesters, and actuators due to its unique role in energy conversion [1]. It is necessary to develop more practical magnetostrictive materials for engineering applications. Up to now, many room-temperature magnetostrictive materials such as rare-earth metals [2], Fe-Ga alloys [3], LaFeSi [4], NiMn-based alloys [5,6,7], and MnCoSi-based alloys have been developed [8,9]. The MnCoSi-based alloys are termed as the most promising candidate owing to their lower cost and higher magnetostrictive coefficient [10]. In addition, MnCoSi-based alloys as a new type of magneto-response material have attracted increasing attention, due to their rich magnetic structure (cycloid type and helical type antiferromagnetic structure, linear ferromagnetic structure) [11,12,13] and rich functional behaviors (magnetocaloric effect [14,15], magnetostrictive effect [8,9], anomalous thermal expansion effect [13,16] and magnetoresistance effect [17,18]).

In recent years, with the development of cryogenic engineering, research on novel cryogenic giant magnetostrictive materials with high efficiency has already aroused scientists’ interest. Previous research manifested that giant magnetostriction up to 7500 ppm and 6000 ppm have been achieved in a Dysprosium single crystal at liquid nitrogen temperature [19,20] and a Fe-32.5Pd single crystal at liquid helium temperature [21], respectively. Nevertheless, the costly process for single crystals hinders their further development. Although polycrystalline alloys are easily prepared, the limited magnetostriction is an obstacle in the path of the application. For example, in polycrystalline FePd alloy, the recoverable magnetostriction is only 635 ppm at 120 K [22]. Therefore, cryogenic giant magnetostrictive materials are desired for their high-potential applications, such as aerospace actuators, cryogenic fluid dynamic converters, and low-temperature transducers.

During past years, many efforts have been made to develop high-performance magnetostrictive materials using MnCoSi-based alloys around room temperature [8,9,23,24,25,26]. The magnetostriction of MnCoSi-based alloys is anisotropy [10,27], and our previous work shows that the MnCoSi alloy with [100]Orth preferred orientation obtained the near linear giant magnetostrictive coefficient around near room temperature [9]. However, the cryogenic giant magnetostriction of MnCoSi has rarely been reported. It is worth mentioning that the meta-magnetic transition from the antiferromagnetic (AFM) state to the ferromagnetic (FM) state in MnCoSi is sensitive to external pressure [28]. Zavorotnev et al. have reported that ferromagnetic occurrence was induced by hydrostatic pressure in the antiferromagnetic region of MnCoSi alloy [29]. Liu et al. have demonstrated that pressure-induced enhanced magnetocaloric effect decreased the critical magnetic field of the MnCoSi alloy [15,28]. Compared under ambient pressure, the maximum magnetic entropy change of MnCoSi alloy at magnetic field 0 ~ 5 T was improved by 35.7% when a 3.2 kbar hydrostatic pressure was applied [15]. The hydrostatic pressure results in the reduction of the critical field for meta-magnetic transition and of the AFM-to-FM transition temperature, which can be ascribed to the reduction of the critical *a*_Orth_-axis and stabilization of the FM state [15,29]. Therefore, hydrostatic pressure is an effective method to adjust the magnetic properties of MnCoSi-based alloy. Inspired by this fact, it is of particular interest to explore the enhanced magnetostriction in MnCo_0.92_Ni_0.08_Si meta-magnetic transition alloys by applying hydrostatic pressure.

Moreover, Johnson and Zhang et al. have reported that when Ni element was employed to replace Co, this adjusted the meta-magnetic transition temperature [30] and magnetostrictive properties [23] of MnCoSi alloy, respectively. It was found that a trace of Ni doping can lead to a significant change in the phase transformation temperature of the alloy [23,30]. In this work, Ni was employed to replace Co in MnCoSi alloy aiming at broadening the transition temperature window and thus obtain a meta-magnetic transition in the vicinity of liquid-nitrogen temperature. Johnson et al. have reported that the competing interactions between antiferromagnetic and ferromagnetic states in MnCo_0.9_Ni_0.1_Si alloy occur at cryogenic temperatures [30]. We chose MnCo_0.92_Ni_0.08_Si, which is near the MnCo_0.9_Ni_0.1_Si alloy composition, for further exploration. In [100]Orth textured MnCo_0.92_Ni_0.08_Si alloy, a giant magnetostrictive effect at cryogenic temperature was achieved by loading hydrostatic pressure, i.e., a large reversible magnetostrictive coefficient (λ) of 5034 ppm has been demonstrated under a magnetic field change of 30,000 Oe at 100 K by applying hydrostatic pressure of 3.2 kbar. In addition, the reversible λ was enhanced from 577 ppm to 2112 ppm at 20,000 Oe and 70 K when a hydrostatic pressure of 6.4 kbar was applied. Thse results show that increasing hydrostatic pressure at low-temperature can result in improved magnetostriction in MnCo_0.92_Ni_0.08_Si alloy.

## 2. Materials and Methods

Polycrystalline MnCo_0.92_Ni_0.08_Si alloy was prepared using high-purity elements Mn, Co, Si, and Ni, by arc-melting technique in an argon atmosphere. The ingot was turned over four times in a copper crucible for homogenization. The sample was sealed into double-sealed quartz capsules with an argon atmosphere of about 0.02 MPa to avoid manganese evaporation. It was treated under a high vacuum for 60 min at 1623 K followed by a multi-stage annealing process [9,16], with a slow cooling rate of 0.2 K/min, then the bulk alloy was obtained. The sample was cut by a diamond saw into 1.0 × 2.0 × 3.0 mm^3^ slices for the strain measurements.

The crystal structure at room temperature was ascertained by powder X-ray diffraction using a Rigaku (Tokyo, Japan) SmartLab X-ray diffractometer, and corresponding XRD patterns were refined by the Rietveld refinement method. The preferred orientation was identified by using XRD on the transverse section of the sample. The microstructural features and the crystallographic orientations were determined by using field-emission-gun SEM (JSM-7001, Jeol, Tokyo, Japan) equipped with an EBSD acquisition camera. The temperature and magnetic field dependences of magnetization were measured by a vibrating sample magnetometer (VSM, VersaLabTM, 30,000 Oe, Quantum Design, San Diego, CA, USA) equipped with the copper-beryllium cylindrical pressure vessel that possesses a maximum pressure of 13 kbar. Daphne 7373 oil was used as the pressure-transmitting medium. The isothermal magnetostriction curves were acquired at different temperatures under various hydrostatic pressures by using a strain gauge (TML Tokyo Sokki Kenkyujo Co., Ltd., Tokyo, Japan) attached to the sample, which was set in the chamber of the Versalab in applied magnetic fields up to 30,000 Oe in the temperature range 70~300 K.

## 3. Results and Discussion

### 3.1. Crystal Structure, Macrostructure and Microstructure

**Figure 1:**
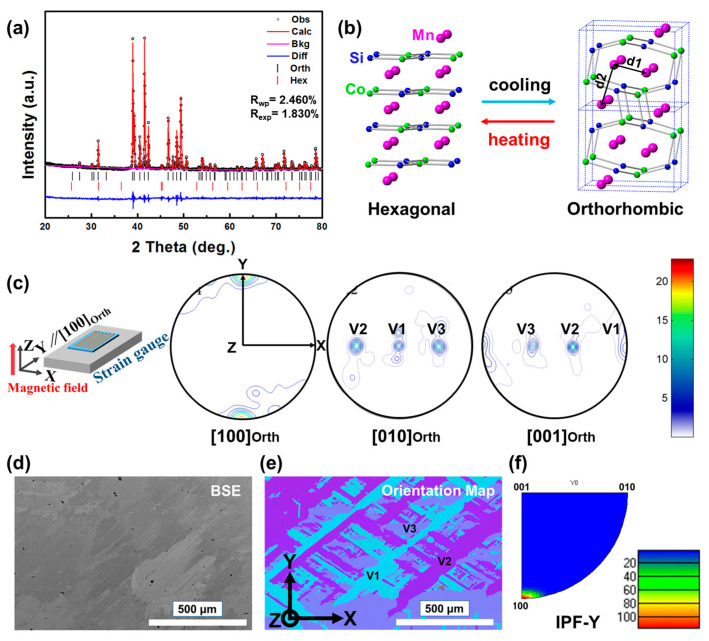
(**a**) Measured (in black circles) and calculated (in red line) powder X-ray diffraction patterns at room temperature for MnCo_0.92_Ni_0.08_Si alloy. The reliability factors (R_wp_ and R_exp_) are indicated in the figure. (**b**) The crystal structure of Hexagonal and Orthorhombic. (**c**) Pole figures of the MnCo_0.92_Ni_0.08_Si alloy were measured by XRD. The inset in (**c**) illustrates the schematic diagram of the specimen. (**d**) Microstructure and (**e**) Orientation Map for the MnCo_0.92_Ni_0.08_Si alloy. (**f**) Inverse pole figure corresponding to Y is calculated according to the EBSD measurements.

Figure 1a shows a representative X-ray diffraction pattern of MnCo_0.92_Ni_0.08_Si powder analyzed by the Rietveld method. According to the refined pattern, the room temperature phase of the alloy can be identified to be a TiNiSi-type orthorhombic structure (*Pnma*, space group #62) and a trace of Ni_2_In-type hexagonal structure (*P6_3_*/*mmc*, space group #194). The refined crystallographic data and the atomic position information are listed in Table 1. It should be noted that in a TiNiSi-type structure of the MnCoSi alloy, each atom occupies 4c position, i.e., (x, 1/4, z), (−x, 3/4, −z), (1/2 − x, 3/4, 1/2 + z) and (1/2 + x, 1/4, 1/2 − z) [11]. It has been reported that the magnetic state of the orthorhombic MnCoSi-based alloy depends on the interatomic distances of the two nearest-neighbor Mn-Mn atoms [10,27], as for the d_1_ and d_2_ annotated in Figure 1b. The MnCo_0.92_Ni_0.08_Si alloy experiences a recoverable martensitic transformation between the honeycomb hexagonal structure and orthorhombic structure [31], as depicted in Figure 1b. To indicate the macro-texture of the sample, [100]Orth, [010]Orth, and [001]Orth pole figures measured by XRD are shown in Figure 1c. From the pole intensity distribution in the [100]Orth pole figure, it can be seen that a strong preferred [100]Orth orientation is parallel to the Y-direction. Such a macroscopic feature is favorable for improving mechanical properties and magnetostriction because of avoiding the inter-stress generated from structure transformation [31] and the mutual compensation of anisotropic magnetostriction [10,27]. Three distinct poles intensities were observed both in [010]Orth and [001]Orth pole figures, indicating there are three martensite variants. Details on the crystallographic relationship between three martensite variants can be found in a previous study [16].

Figure 1d shows the room-temperature microscopic microstructure of the MnCo_0.92_Ni_0.08_Si alloy, covering the region of 1.2 mm × 1.0 mm. Consistent with our previous observations [9,16], the original hexagonal phase develops coarse grains with several millimeters in average diameter, due to the grain growth during the annealing process at 1273 K for 48 h. According to the orientation map (Figure 1e), three main plate colors mean there are three equivalent martensite variants (V1, V2, and V3). Martensite variants plates with different crystallographic orientations are assembled by inter-plates interfaces. Figure 1f shows the inverse pole figure corresponding to the Y-direction calculated from the EBSD measurements. It can be found that the martensite plates form the [100]Orth preferred orientation along the Y-direction, which is consistent with the results obtained in the macro-texture measurement. At the same time, the actual compositions of the present MnCo_0.92_Ni_0.08_Si alloy examined by energy dispersive spectrometry (EDS) are Mn_0.98_Co_0.91_Ni_0.09_Si_1.02_. The experimentally determined composition of each alloy is verified to be close to the designed one.

### 3.2. Magnetic Properties

**Figure 2:**
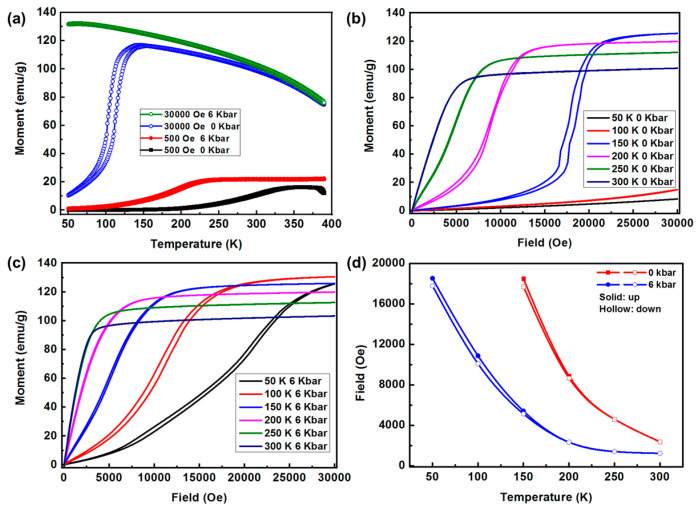
(**a**) The thermomagnetic loops (M-T) were measured upon heating and cooling with 0 kbar and 6 kbar for MnCo_0.92_Ni_0.08_Si alloy at the magnetic field of 500 Oe and 30,000 Oe. Field-up and field-down isothermal magnetization curves (M-H) within the magnetic range between 0~30,000 Oe (**b**) ambient pressure and (**c**) 6 kbar. (**d**) The temperature dependence of critical fields for driving meta-magnetic transition field-up and field-down processes at different hydrostatic pressure derived from (**b**,**c**).

The magnetization as a function of temperature for MnCo_0.92_Ni_0.08_Si, at ambient pressure (0 kbar) and the hydrostatic pressure (6 kbar), measured during heating and cooling in the presence of 500 Oe and 30,000 Oe magnetic field, are shown in Figure 2a. It should be noted that the direction of the magnetic field is parallel to the Z-direction of the sample, as shown in Figure 1c. Under ambient pressure and a magnetic field of 500 Oe, the magnetization of the sample is very low (<1 emu/g), which indicates that the antiferromagnetic exchange is strong and the sample is in an AFM state below 200 K. With increasing temperature, a smooth meta-magnetic transition from a helical AFM state to an ordered FM state is observed, indicating that the sample exhibits FM state at room temperature. In addition, while the temperature is higher than 380 K, the decrease of magnetization indicates that a part of the alloy undergoes a ferromagnetic to paramagnetic (FM-PM) transition with further increasing temperature [30]. Increasing hydrostatic pressure to 6 kbar, the magnetization begins to increase at around 100 K during the heating process, which means that the meta-magnetic transition occurs at a lower temperature. It is also notable that the saturation magnetization of the sample under high hydrostatic pressure appears to be slightly higher than that under ambient pressure at the same temperature, which can be ascribed to the enhanced ferromagnetic exchange due to high pressure-induced unit cell shrinkage. While the applied external magnetic field was increased to 30,000 Oe, the meta-magnetic transition occurred sharply in the vicinity of 120 K, as shown in Figure 2a. On this basis, increasing the hydrostatic pressure to 6 kbar can further widen the lower limit of the FM state temperature region to 50 K, with little change in the high temperatures region. This means that the meta-magnetic transition of the MnCo_0.92_Ni_0.08_Si alloy can be induced at a lower temperature when the sample was subjected to hydrostatic pressuring. In general, the meta-magnetic transition would be modified by external hydrostatic pressure since AFM-FM competition has an intimate relationship with the lattice parameters of MnCoSi-based alloys and pressure-induced unit cell shrinkage is inevitable [15].

Figure 2b,c present the isothermal magnetization (M-H) curves of MnCo_0.92_Ni_0.08_Si at ambient pressure and selected hydrostatic pressure (6 kbar), respectively. At 50 K and 100 K, the magnetization varies almost linearly under field up to 30,000 Oe with little increase under ambient pressure, indicating the alloy maintains an AFM state. As the temperature increased to 150 K, a meta-magnetic transition from AFM to FM is observed, exhibiting a sharp increase over a critical magnetic field (H_cr_, defined as a magnetic field corresponding to 50% of saturation magnetization [8]) followed by saturation. There is noticeable hysteresis between field-up and field-down plots. Furthermore, the magnetic hysteresis was decreased with increasing temperature, indicating that the phase transition changed from the first-order transition to the second-order transition. When the sample was subjected to hydrostatic pressure (6 kbar), Figure 2c reveals that the meta-magnetic transition can be activated at a lower temperature (50 K) by the maximum magnetic field of 30,000 Oe and the H_cr_ was reduced. The results are consistent with the observation of the M-T curves, that is, the external stress field facilitates the FM state in MnCo_0.92_Ni_0.08_Si, and thus lowered meta-magnetic transition temperature and critical field were obtained. In contrast to the sharp transition depicted in Figure 2b, the meta-magnetic transition under high pressure in Figure 2c is broad and smooth, which can be ascribed to the strong competition between AFM and FM states. In addition, Figure 2d summarizes the critical field (H_cr_) as a function of temperature for MnCo_0.92_Ni_0.08_Si alloy at different hydrostatic pressure (0 kbar and 6 kbar). One can see that H_cr_ decreases significantly with increasing external pressure. At the same temperature, a smaller meta-magnetic transition critical field was needed and narrower magnetic hysteresis was observed with increasing hydrostatic pressure. For example, at 150 K, while the hydrostatic pressure was increased from 0 kbar to 6 kbar, the critical field decreased from 18,000 Oe to 5200 Oe, and the hysteresis disappears.

### 3.3. Magnetostriction

**Figure 3:**
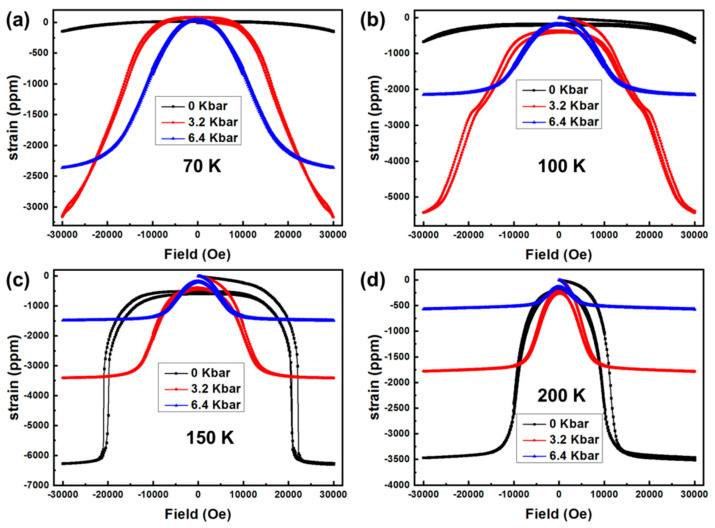
Isothermal magnetostriction under various pressure (**a**) 70 K, (**b**) 100 K, (**c**) 150 K and (**d**) 200 K.

Figure 3 gives a comparison of the magnetic field dependence of magnetostriction for the MnCo_0.92_Ni_0.08_Si alloy measured under various hydrostatic pressures at selected temperatures. It should be noted that the strain values along the [100]Orth were measured and the magnetic field was applied parallel to the [010]Orth direction, as shown a schematic diagram in Figure 1c. There is an obvious difference in the saturation magnetostriction for the three pressure states. At 70 K without external hydrostatic pressure, the magnetostrictive coefficient changes little when the magnetic field increases from 0 Oe to 30,000 Oe (Figure 3a). However, compared with ambient pressure, the saturation magnetostrictive coefficient is up to 3159 ppm and 2356 ppm for 3.2 kbar and 6.4 kbar, respectively, which is related to the promotion of the induced FM state at higher hydrostatic pressure. The subsequent fall of the magnetostriction for the MnCo_0.92_Ni_0.08_Si alloy under 6.4 kbar is a result of field-induced partially meta-magnetic transition as pressure stabilizes the FM state. It should be noted, however, that at 70 K and with a small magnetic field (<20,000 Oe), the magnetostriction of MnCo_0.92_Ni_0.08_Si crystal still increases with the hydrostatic pressure. For example, the magnetostrictive coefficients reach 1734 ppm and 2112 ppm under 3.2 kbar and 6.4 kbar, respectively, at 20,000 Oe, which is comparable to the Terfenol-D compound [32]. Similar experimental results were also observed at 100 K, as shown in Figure 3b. These details prove that the hydrostatic pressure is favorable to cryogenic giant magnetostriction under a small magnetic field. Noteworthy is that magnetostriction cannot completely recover after the first cycle but becomes fully recoverable in the following cycles (Figure 3b–d).

Raising the temperature to 150 K, we recorded the magnetostrictive effect at different pressures, as illustrated in Figure 3c. At ambient pressure, it can be seen that the magnetostriction began to increase rapidly when the magnetic field was increased to a certain value (~20,000 Oe), at which the field-induced meta-magnetic transition was completed. In contrast, after slightly increasing the external pressure to 3.2 kbar, the MnCo_0.92_Ni_0.08_Si alloy reached saturation magnetostriction around 2826 ppm at 15,000 Oe, indicating a significant enhancement of magnetostrictive coefficient under a small magnetic field compared with no external pressure. Further increasing hydrostatic pressure to 6.4 kbar, the saturation magnetostriction decreases down to 1260 ppm as a result of the stress-induced almost complete FM states.

Figure 3d depicts the magnetostriction curves at 200 K for MnCo_0.92_Ni_0.08_Si alloy versus hydrostatic pressure. Without hydrostatic pressure, the alloy undergoes easy saturation magnetostriction of 3340 ppm compared with the values at lower temperatures. A similar case has also been detected in the M-H curves shown in Figure 2c as well. However, the saturation magnetostriction is suppressed by hydrostatic pressure at 200 K, i.e., 1550 ppm and 439 ppm for 3.2 kbar and 6.4 kbar, respectively. Nonetheless, a slight modification of hydrostatic pressure of 3.2 kbar causes an impressive enhancement in magnetostrictive coefficient when the magnetic field is below 9000 Oe.

It should be noted that in our previous work [9], the results show that the magnetostriction of MnCoSi alloys by applying a magnetic field of 50,000 Oe only occurs above 240 K, and that at temperatures below 210 K, 50,000 Oe is too small to active the meta-magnetic transition. However, in the present work, it is demonstrated that the magnetostrictive effect of MnCo_0.92_Ni_0.08_Si alloy, which resulted from meta-magnetic transition, could be induced when the sample was subjected to a magnetic field of 30,000 Oe at 150 K. In addition, large cryogenic temperature magnetostriction can be obtained in MnCo_0.92_Ni_0.08_Si alloy by applying hydrostatic pressure.

The experimental results in this work show that the magnetization of the MnCo_0.92_Ni_0.08_Si alloy is increased by hydrostatic pressure, which indicates that the meta-magnetic transition can be driven by a lower magnetic field. This is consistent with the results reported in previous studies about MnCoSi alloy subjected to hydrostatic pressure [15,28]. According to the previous reports [10,27] on the evolution of the lattice parameters of MnCoSi-based alloys with the magnetization states, it was found that the nearest neighbor Mn-Mn distances, i.e., d_1_ and d_2_, changed in the opposite direction (d_1_ increases and d_2_ shrinks) during the process of non-collinear antiferromagnetic transition to ferromagnetic states. Therefore, it can be speculated that the influence of hydrostatic pressure on d_1_ and d_2_ of MnCo_0.92_Ni_0.08_Si alloy is different, that is to say, d_1_ increase and d_2_ decrease with increasing hydrostatic pressure. At the same time, considering that changes in the Mn-Mn separations act as a precursor to the magnetostrictive effect in MnCoSi-based alloys [9,10,27], enhanced magnetostriction can be obtained in MnCo_0.92_Ni_0.08_Si alloy under a hydrostatic pressure environment. It can be seen that magnetostrictive values are quite sensitive to hydrostatic pressure, according to Figure 3.

**Figure 4:**
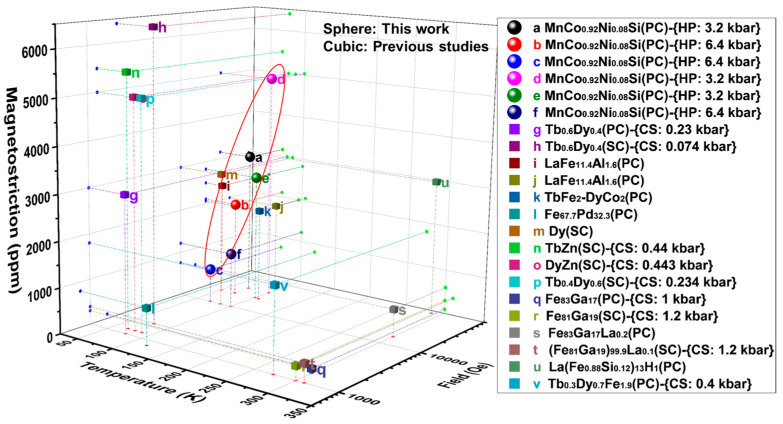
Comparison of magnetostrictive values (λ) between the present alloy and some other magnetostrictive materials under single/multiple external fields: g [33], h [34], i [35], j [35], k [32], l [22], m [19], n [36], o [37], p [38], q [39], r [3,40], s [41,42], t [3], u [4], v [43]. (The abbreviations PC, SC, HP, and CS denote polycrystalline, single crystalline, hydrostatic pressure, and compress stress).

To compare with others, the reversible magnetostrictive coefficients obtained from the present [100]Orth-oriented MnCo_0.92_Ni_0.08_Si alloy as well as some other magnetostrictive materials reported in the previous investigation are summarized in Figure 4 [3,4,19,22,32,33,34,35,36,37,38,39,40,41,42,43]. It is demonstrated that the magnetostriction obtained in the present work is comparable to the rare earth compound single-crystalline materials [19,34,36,37,38], and larger than the magnetostrictive values of polycrystalline materials around liquid nitrogen temperature [32,35].

## 4. Conclusions

In summary, applying hydrostatic pressure could change nearest neighbor Mn-Mn distances, i.e., extend d_1_ and shorten d_2_, thus strengthening the magnetization of MnCo_0.92_Ni_0.08_Si alloy, then reducing the critical field of meta-magnetic transition and lowering the meta-magnetic transition temperature. Since the FM state can be stabilized by hydrostatic pressure, a large cryogenic magnetostriction has been successfully obtained in MnCo_0.92_Ni_0.08_Si meta-magnetic transition alloy. As a result, after applying the hydrostatic pressure of 3.2 kbar, giant magnetostriction of 5034 ppm at 100 K and 30,000 Oe was realized in an [100]_Orth_ textured MnCo_0.92_Ni_0.08_Si alloy. The obtained magnetostriction under 20,000 Oe is close to 2112 ppm at 70 K and 6.4 kbar. This work indicates that the MnCoSi-based alloys appear to be a potential candidate material for low-temperature engineering, and also enriches the investigation of the magnetostrictive effect under multiple fields.

## Figures and Tables

**Table 1:** Experimental details and refined crystallographic data for the MnCo_0.92_Ni_0.08_Si alloy.

Parameters	MnCo_0.92_Ni_0.08_Si
*a*_orth_ (Å)	5.8465
*b*_orth_ (Å)	3.6843
*c*_orth_ (Å)	6.8662
*V* (Å^3^)	147.8997
*x*_Mn_/*z*_Mn_	0.0229/0.1815
*x*_Co(Ni)_/*z*_Co(Ni)_	0.1552/0.5593
*x*_Si_/*z*_Si_	0.7699/0.6263
d_1_/d_2_ (Å)	3.110/3.071
R_wp_/R_exp_ (%)	2.460/1.830

## Data Availability

The raw/processed data required to reproduce these findings cannot be shared at this time as the data also comprise a part of an ongoing study.
